# Safety and efficacy of lobaplatin combined with 5-fluorouracil as first-line induction chemotherapy followed by lobaplatin-radiotherapy in locally advanced nasopharyngeal carcinoma: preliminary results of a prospective phase II trial

**DOI:** 10.1186/s12885-017-3080-4

**Published:** 2017-02-15

**Authors:** Liang-Ru Ke, Wei-Xiong Xia, Wen-Ze Qiu, Xin-Jun Huang, Jing Yang, Ya-Hui Yu, Hu Liang, Guo-Ying Liu, Yan-Fang Ye, Yan-Qun Xiang, Xiang Guo, Xing Lv

**Affiliations:** 1 Department of Nasopharyngeal Carcinoma, Sun Yat-Sen University Cancer Center, 651 Dongfeng Road East, Guangzhou, Guangdong 510060 China; 2 State Key Laboratory of Oncology in Southern China, Collaborative Innovation Center for Cancer Medicine, Guangzhou, Guangdong 510060 China

**Keywords:** Lobaplatin, Nasopharyngeal carcinoma, Locally advanced, First-line, Chemotherapy

## Abstract

**Background:**

Due to improvements in imaging and radiological techniques as well as the use of chemotherapy, distant metastasis has become the predominant mode of treatment failure in patients with locally advanced nasopharyngeal carcinoma (LA-NPC). Platinum-based systemic chemotherapy has shown survival benefits and is now the standard strategy for systemic therapy in patients with LA-NPC. Notably, the third-generation platinum reagent lobaplatin has shown anti-tumor effects in several solid tumors with lower incidences of gastrointestinal, hepatic and renal toxicity relative to other platinum drugs. However, the safety and efficacy of lobaplatin as a first-line regimen in patients with LA-NPC are undetermined.

**Methods:**

Patients with stage III–IVa-b NPC received lobaplatin at a dose of 30 mg/m2 on days 1 and 22 combined with a continuous 120-h intravenous injection of 5-fluorouracil at a dose of 4 g/m2 followed by lobaplatin at a dose of 50 mg/m2 on days 43 and 64 concomitant with intensity-modulated radiation therapy. Objective response rates and acute toxicity were assessed based on RECIST (1.1) and CTCAE v.3.0, respectively. Kaplan-Meier analysis was used to calculate survival rates.

**Results:**

Fifty-nine patients were enrolled, and 44 patients (74.6%) received allocated cycles of chemotherapy. The objective response rates were 88.1% (95% confidence interval [CI], 0.77 to 0.95) and 100% after induction chemotherapy (ICT) and concurrent chemoradiotherapy (CRT), respectively. With a median follow-up period of 44 months, the 3-year estimated progression-free survival and overall survival were 86.4% (95% CI, 69.8 to 98.8) and 94.9% (95% CI, 89.5 to 100), respectively. The most common grade 3–4 toxicities were neutropenia (8.5%) and thrombocytopenia (40.7%) after ICT and CRT, respectively.

**Conclusion:**

Lobaplatin combined with 5-fluorouracil followed by lobaplatin-RT treatment showed encouraging anti-tumor effects with tolerable toxicities in patients with LA-NPC. Randomized controlled trials of lobaplatin in patients with LA-NPC are warranted.

**Trial registration:**

This trial was registered with the Chinese Clinical Trials Registry and approved on March 31^st^, 2012, number ChiCTR-ONC-12002060.

## Background

Nasopharyngeal carcinoma (NPC), a malignancy derived from epithelial cells of the nasopharynx, is endemic in Southern China and Southeast Asia [1]. The development of high-resolution imaging and radiological techniques has increased the local control rate of NPC up to 95% [2], leaving distant metastasis as the major cause of treatment failure. To address this issue, a combined regimen of chemotherapy and radiotherapy has been recommended as the standard treatment strategy for locally advanced NPC (LA-NPC) [3]. However, the survival benefits of sequential chemotherapy for NPC patients are still controversial [4–8]. Induction chemotherapy (ICT) can improve both progression-free survival (PFS) and/or overall survival (OS) [8–14]. Ma J et al recently demonstrated that the introduction of cisplatin, fluorouracil, and docetaxel (TPF) induction chemotherapy to concurrent chemotherapy could significantly improve the PFS of patients with LA-NPC [15], while additional randomized controlled clinical trials to determine the role of systemic chemotherapy in NPC during the intensity modulation radiotherapy (IMRT) era are still underway.

A platinum-based regimen is recommended as the first-line chemotherapeutic strategy for NPC according to the NCCN guidelines. Cisplatin, the first anti-cancer reagent, has shown encouraging anti-tumor efficacy in NPC both alone and in combination with other treatments [3, 5, 16, 17]. However, cisplatin can induce severe side effects, including dose-limiting nephrotoxicity, cumulative peripheral sensory neuropathy, ototoxicity and gastrointestinal reactions, such as nausea or vomiting, which can reduce patient compliance for chemotherapy administration or even cause treatment interruptions [18]. Moreover, the requirement of a massive fluid infusion of cisplatin to avoid renal toxicity extends the inpatient period and limits the application of this treatment in patients with heart and renal dysfunction. Lobaplatin, a third-generation platinum reagent, forms DNA adducts and induces DNA damage, resulting in cell apoptosis, which is similar to the pharmacological mechanisms of other platinum reagents [19]. Lobaplatin has shown robust anti-tumor efficacy in multiple solid tumors, such as breast cancer, hepatocellular carcinoma, non-small cell lung cancer, transitional cell carcinoma, ovarian cancer and cervical squamous carcinoma [20–23], but without the above-listed toxicities. Moreover, lobaplatin has no multidrug resistance with other platinum drugs, such as cisplatin or carboplatinum [20]. Though lobaplatin costs approximately 16-fold more than cisplatin, there is no increased economic burden to patients due to the high insurance coverage of both reagents. Major characteristics of cisplatin and lobaplatin are compared in Table 1.

**Table 1:** Comparison of cisplatin and lobaplatin

Category	Cisplatin	Lobaplatin
Product generation	First generation	Third generation
Chemical structure		
Anti-tumor mechanism	Forms DNA-drug adducts, resulting in DNA damage and cell apoptosis	
Half-life period	Over 24 h (total platinum) [33], mainly metabolized through the kidney	131 ± 15 min (free platinum) and 6.8 ± 4.3 days (total platinum) [19], mainly metabolized through the kidney
Anti-tumor spectrum	Ovarian, testicular, bladder, colorectal, lung, head and neck cancer	Metastatic breast cancer, chronic myelogenous leukemia, and small cell lung cancer
Side effects	Nephrotoxicity, cumulative peripheral sensory neuropathy, ototoxicity, nausea and vomiting	Thrombocytopenia
Additional medication	Hydration for high dose	No
Solvent	Normal saline or glucose	Glucose
Drug resistance	Easy to produce	Rare and no cross-resistance with cisplatin
Expense per cycle for platinum drugs	¥160.0-200.0 ($23.8-29.8)	¥2800.0-3400.0 ($417.9-507.5)

A combination of lobaplatin and docetaxel has exhibited particularly effective anti-tumor activity in recurrent and metastatic NPC [24]. Herein, to further determine the anti-tumor efficacy of lobaplatin in LA-NPC, we performed a phase II clinical trial to determine the safety and efficacy of lobaplatin combined with 5-fluorouracil (5-FU) as a first-line ICT strategy followed by lobaplatin-radiotherapy (lobaplatin-RT) in LA-NPC.

## Methods

### Aim of study and end points

The main purpose of this trial was to test the feasibility and efficacy of delivering lobaplatin combined with 5-FU as a first-line ICT regimen followed by lobaplatin-RT in patients with LA-NPC. The primary end point was the objective response rate (ORR), and secondary end points included overall survival (OS), progression-free survival (PFS), distant metastasis-free survival (DMFS) and local recurrence-free survival (LRFS). Acute toxicities during ICT and lobaplatin-RT were also observed. The study was designed as a single arm, open-labeled phase II study.

### Participant enrollment and characteristics

Regular evaluations were performed for all enrolled participants as previously described [2]. These included medical history, physical examination, pathology diagnosis, electrocardiogram, and laboratory testing, including but not limited to liver and kidney function and Epstein Barr virus (EBV) DNA copy number assessment. To ensure tumor margins were well-defined, magnetic resonance imaging (MRI) of the nasopharynx and neck were conducted in all patients, except those with contraindications (e.g., pacemaker or stent); for these cases, computed tomography (CT) was used. Regular work-ups including chest X-rays, abdominal sonography and bone scans, or positron emission tomography/computed tomography (PET/CT) as an optional substitution to evaluate distant metastases, were also performed based on patient preference and financial capacity. The patients were staged according to the American Joint Committee on Cancer (AJCC, 7^th^ edition) staging system based on the above imaging results.

The following inclusion criteria were applied for enrollment: untreated patients aged 18 to 60 years; stage III or IVa-b disease; histopathologically confirmed WHO type II or III NPC; leukocytes ≥ 4.0X10E9; neutrophils ≥ 1.5X10E9; platelets ≥ 100X10E9; hemoglobin ≥ 90 g/L; aminotransferase ≤ 2XUNL; serum creatinine ≤ 1.5XUNL; and no evidence of dysfunction in important organs (e.g., heart, lung, liver and kidney) or other malignancies. All participants provided informed consent. Patients who were known or suspected to be allergic to cisplatin, who had uncontrolled infection or a physical disease that could not tolerate chemotherapy/radiotherapy, or who could not coordinate contact for follow-up were excluded. Additionally, pregnant females or patients who underwent immune repressive treatment such as that following organ transplantation were also not admitted. Patients who could not tolerate the treatment toxicities or failed to receive planned treatment due to voluntary refusal or a physician’s decision were withdrawn from the study.

From April 1, 2012 to Oct 31, 2012, 59 patients with confirmed undifferentiated nonkeratinizing NPC were enrolled in this study. Their demographic and clinical characteristics before treatment are listed in Table 2. The median age of the participants was 43 years. Forty-three (72.9%) males and 16 (27.1%) females were enrolled. Twenty-two participants (37.3%) had a high serum EBV DNA copy number (>4000 copies/ml) before treatment. Twenty-nine (49.2%) participants with stage III disease and 30 (50.8%) participants with stage IV_a-b_ disease were enrolled.

**Table 2:** Distribution of patient demographics and clinical characteristics before treatment

Characteristics	Patients	
	No.	%
Age, years		
Median	43	
Range	19-59	
Sex		
Male	43	72.9
Female	16	27.1
Histology, WHO type^a^		
III	59	100.0
EBV DNA copy no. (pre-treatment)		
Low (≤4000 copies/ml)	32	54.2
High (>4000 copies/ml)	22	37.3
NA	5	8.5
T stage^b^		
2	4	6.8
3	30	50.8
4	25	42.4
N stage^b^		
0	3	5.1
1	26	44.1
2	23	39.0
3	7	11.9
Clinical stage^b,c^		
III	29	49.2
IVa	23	39.0
IVb	7	11.9
ECOG score		
0	3	5.1
1	56	94.9

*Abbreviation ECOG* Eastern Cooperative Oncology Group; *NA* not available

^a^III, undifferentiated nonkeratinizing carcinoma

^b^According to the 7^th^ edition AJCC staging system

^c^III, T3N0-2 M0, T1-2N2M0; IVa, T4N0-2 M0; IVb, T1-4N3M0

### Treatment

Allocated treatments including two cycles of ICT followed by two cycles of concomitant chemotherapy and radical intensity-modulated radiotherapy (IMRT) to the nasopharynx and neck were delivered to all patients. The ICT consisted of 30 mg/m^2^ lobaplatin (intravenous infusion, day 1) and 4 g/m^2^ 5-fluorouracil (continuous 120-h intravenous injection) during each cycle. The concurrent chemotherapy consisted of 50 mg/m^2^ lobaplatin (intravenous infusion, day 1) during each cycle. The interval of cycles was 3 weeks.

Radical IMRT was initiated on the same day as first concomitant chemotherapy. RT prescription doses of 68–70 Gy, 62–68 Gy and 54–60 Gy were delivered to the planning target volume (PTV) of the primary nasopharynx tumor, involved cervical lymph nodes and lymph node-negative areas, respectively. All patients received a regular fraction during RT, five fractions per week and 30–33 fractions in total.

National Cancer Institute (NCI) Common Toxicity Criteria (CTC) V.3.0 were used to determine acute adverse events. The prescription dose of chemotherapy was modified based on the toxicity induced by the previous chemotherapy cycle and was decreased to 75% of the allocated dose when a patient suffered from any of the following toxicities: granulocytopenia fever, platelet count ≤ 25,000/μL, or grade 3 nausea and/or vomiting. The prescription dose of chemotherapy was decreased to 50% of the planned dose when a patient developed any of the following toxicities: grade 4 or greater nausea and/or vomiting or creatinine clearance around 35–49 ml/min. Prophylactic administration of hematopoietic colony-stimulating factor was performed on days 3–8 of the next chemotherapy cycle in the patients who developed grade 4 granulocytopenia. Chemotherapy was interrupted when patients developed grade 4 hematological, hepatic or kidney toxicity and was administered again if the toxicity decreased to grade 2 or less. Chemotherapy was no longer delivered to patients with a remitted time of over 2 weeks.

**Fig. 1:**
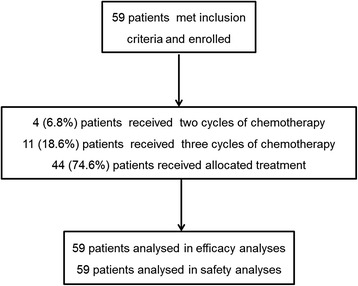
Flowchart of the trial

Of the 59 participants, 44 (74.6%) completed all cycles of planned chemotherapy, 11 (18.6%) received three cycles of chemotherapy, and four (6.8%) received only two cycles of chemotherapy (Fig. 1).

### Assessment of efficacy and follow-up

Efficacy was assessed after two cycles of ICT, after concurrent chemoradiotherapy (CRT) and 3 months after CRT. Treatment response was evaluated based on Response Evaluation Criteria In Solid Tumors (RECIST 1.1). The follow-up frequency for the first 2 years and the 3^rd^ to 5^th^ years was 3 and 6 months, respectively. Regular examinations including physical examinations, indirect nasopharyngoscopy, fiber nasopharyngoscopy, and MRI of the nasopharynx and neck were used for efficacy evaluation and follow-up. Patients with confirmed local recurrence (LR) were diagnosed via MRI and biopsy. Diagnosis of distant metastasis (DM) was confirmed by a regular workup, including CT, MRI, bone scan or PET/CT, and needle biopsy when available. Salvage treatments including re-irradiation, surgery and/or ablation to local lesion and/or systemic chemotherapy were performed in patients with LR or DM as long as they had the indications.

### Statistical analysis

SPSS 22.0 (SPSS, Chicago, IL) was used to perform all analyses in this study. A Simon Two-Stages Design was used to determine the sample size. We assumed the ORR to be 80%, and less than 60% was unacceptable. The estimated rate of loss to follow-up was 10%. We set the power to detect the effectiveness of the treatment strategy in our study as 0.9 with a two-sided significance of *P* = 0.05. Accordingly, a total of 59 evaluable patients were required. OS, PFS, LRFS and DMFS were calculated using Kaplan-Meier analysis. All time-to-event end points were calculated from the first date of treatment to the date of treatment failure or the last day of follow-up. Patients who developed either DM or LR were followed up until death or until the last scheduled day of follow-up. All efficacy analyses were performed in the intention-to-treat population. All patients who received at least one cycle of chemotherapy were included in the toxicity analysis. This trial was registered with the Chinese Clinical Trials Registry, number ChiCTR-ONC-12002060.

## Results

### Assessment of treatment efficacy

Fifty-two (88.1%) participants experienced an objective response (OR) after two cycles of ICT, with eight (13.6%) and 44 (74.6%) participants experiencing a complete response (CR) and a partial response (PR), respectively. After a complete course of treatment, all (100%) patients experienced an OR, with 47 (79.7%) and 12 (20.3%) participants experiencing a CR and PR, respectively. Of these 12 participants, seven and four individuals experienced residual disease in the cervical lymph nodes or nasopharynx. One patient had persistent disease in both sites after CRT. Three months after CRT, 51 (86.4%) and two (3.4%) patients experienced CR and PR, respectively, with six patients being lost to follow-up at this time (Table 3). Of the two patients who experienced PR 3 months after CRT, one had residual disease in the nasopharynx and experienced CR 6 months after CRT and the other had persistent and stable disease in the cervical lymph nodes during the close follow-up.

**Table 3:** Treatment responses in 59 patients

Treatment response	After two cycles of ICT No. (%)	After CRT No. (%)	Three months after CRT No. (%)
Complete response (CR)	8/59(13.6)	47/59(79.7)	51/59(86.4)
Partial response (PR)	44/59(74.6)	12/59(20.3)	2/59(3.4)
Stable disease (SD)	4/59(6.8)	0/59(0)	0/59(0)
Not available	3/59(5.1)	0/59(0)	6/59(10.2)
Objective response (CR + PR)	52/59(88.1)	59/59(100)	53/59(89.8)
95% CI of ORR	(0.77, 0.95)	NA	(0.82, 0.98)

*Abbreviation ICT* induction chemotherapy; *CRT* concurrent chemoradiotherapy; *CI* confidence interval; *ORR* objective response rate; *NA* not available

### Failure patterns

Over a median follow-up time of 44 months (range from 1 to 47 months), eight (13.6%) patients experienced disease progression, with a range of progression time of 11 to 31 months. Of these patients, two (3.4%) developed local relapse, one in the nasopharynx and the other in the cervical lymph nodes. Moreover, six (10.2%) patients developed DM. The sites of DM were the liver (*n* = 1), the lung (*n* = 3) and multiple organs (*n* = 2). Three (5.1%) patients with DM died of cancer progression, and the OS time ranged from 1 to 15 months.

**Fig. 2:**
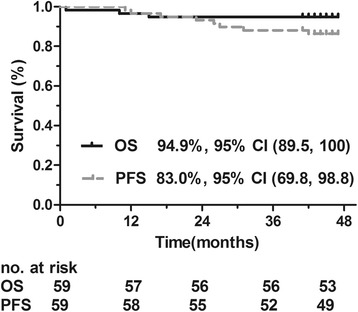
Overall survival (OS) and progression-free survival (PFS) rates in patients with locally advanced nasopharyngeal carcinoma treated with lobaplatin-fluorouracil followed by lobaplatin-radiotherapy

**Fig. 3:**
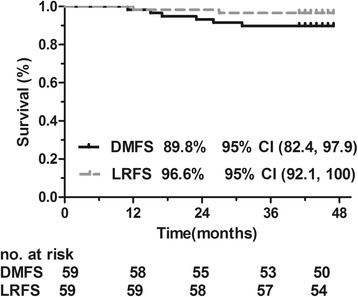
Distant metastasis-free survival (DMFS) and local recurrence-free survival (LRFS) rates in patients with locally advanced nasopharyngeal carcinoma treated with lobaplatin-fluorouracil followed by lobaplatin-radiotherapy

The 3-year estimated survival rates and the 95% confidence intervals (CIs) for all time-to-event end points are listed in Table 4. Kaplan-Meier survival curves for OS or PFS and DMFS or LRFS are shown in Figs. 2 and 3. The median of all time-to-event end points had not been reached until after the paper was published.

**Table 4:** Three-year estimates of time-to-event end points

End point	3-Year estimate (%)	95% Confidence interval
Progression-free survival	83.0	(69.8, 98.8)
Overall survival	94.9	(89.5, 100)
Local recurrence-free survival	96.6	(92.1, 100)
Distant metastasis-free survival	89.8	(82.4, 97.9)

### Acute adverse events

During the ICT, the following grade 3–4 acute adverse events occurred in descending order: neutropenia (*n* = 5, 8.5%), leucopenia (*n* = 4, 6.8%), thrombocytopenia (*n* = 3, 5.1%), hepatotoxicity (*n* = 2, 3.4%) and stomatitis (*n* = 1, 1.7%). Likewise, during the CRT, the main grade 3–4 acute adverse events included thrombocytopenia (*n* = 24, 40.7%), leucopenia (*n* = 20, 33.9%), neutropenia (*n* = 15, 25.4%), anemia (*n* = 10, 16.9%), stomatitis (*n* = 2, 3.4%) and hepatotoxicity (*n* = 2, 3.4%). One (1.7%) and two (3.4%) patients developed grade 4 neutropenia during ICT and CRT, respectively. Eight (13.6%) patients developed grade 4 thrombocytopenia after the complete treatment course (Table 5). The duration time of grade 3–4 thrombocytopenia is also listed in Table 5. In addition, among the 15 patients who received only two or three cycles of chemotherapy, chemotherapy was interrupted in 12 and three patients due to persistent thrombocytopenia or leucopenia, respectively, although hematopoietic colony-stimulating factor was administered once myelosuppression occurred.

**Table 5:** Acute adverse events in 59 patients

Adverse events	No. (%) of patients by toxicity grade during ICT				No. (%) of patients by toxicity grade during CRT			
	1	2	3	4	1	2	3	4
Hematological								
Anemia	28(47.5)	4(6.8)	0(0)	0(0)	22(37.3)	13(22.0)	10(16.9)	0(0)
Neutropenia	17(28.8)	17(28.8)	4(6.8)	1(1.7)	13(22.0)	22(37.3)	13(22.0)	2(3.4)
Leucopenia	20(33.9)	14(23.7)	4(6.8)	0(0)	14(23.7)	20(33.9)	20(33.9)	0(0)
Thrombocytopenia	9(15.3)	10(16.9)	3(5.1)	0(0)	4(6.8)	20(33.9)	16(27.1)	8(13.6)
Days of grade 3 or greater thrombocytopenia					≤7	8-14	≥15	
					17(28.8)	6(10.2)	1(1.7)	
Non-hematological								
Allergy	0(0)	0(0)	0(0)	0(0)	0(0)	0(0)	0(0)	0(0)
Weight loss	5(8.5)	0(0)	0(0)	0(0)	33(55.9)	5(8.5)	0(0)	0(0)
Stomatitis (mucositis)	2(3.4)	1(1.7)	1(1.7)	0(0)	49(83.1)	8(13.6)	2(3.4)	0(0)
Nausea	21(35.6)	2(3.4)	0(0)	0(0)	14(23.7)	6(10.2)	0(0)	0(0)
Vomiting	9(15.3)	2(3.4)	0(0)	0(0)	7(11.9)	6(10.2)	0(0)	0(0)
Diarrhea	1(1.7)	0(0)	0(0)	0(0)	1(1.7)	0(0)	0(0)	0(0)
Hepatotoxicity	21(35.6)	3(5.1)	2(3.4)	0(0)	23(39.0)	3(5.1)	2(3.4)	0(0)
Nephrotoxicity	10(16.9)	0(0)	0(0)	0(0)	11(18.6)	0(0)	0(0)	0(0)
Cardiotoxicity	0(0)	0(0)	0(0)	0(0)	0(0)	0(0)	0(0)	0(0)
Ototoxicity	0(0)	0(0)	0(0)	0(0)	10(16.9)	0(0)	0(0)	0(0)
Neurotoxicity	0(0)	0(0)	0(0)	0(0)	0(0)	0(0)	0(0)	0(0)
Joint and muscular ache	0(0)	0(0)	0(0)	0(0)	0(0)	0(0)	0(0)	0(0)
Alopecia	1(1.7)	0(0)	0(0)	0(0)	59(100)	0(0)	0(0)	0(0)

*Abbreviation ICT* induction chemotherapy; *CRT* concurrent chemoradiotherapy

## Discussion

Presently, concurrent chemotherapy with or without ICT is the standard treatment strategy for patients with LA-NPC [25]. Although the survival benefits of ICT have been inconsistent across prior studies, ICT can theoretically shrink local regional tumors and eradicate micrometastases, resulting in reduced RT volume, decreased RT dose needed for organs at risk and a slower rate of DM. Moreover, a prior study showed that changing from adjuvant cisplatin and 5-fluorouracil to induction cisplatin and capecitabine caused a favorable trend of increasing efficacy with less toxicity in LA-NPC [8]. Similarly, several phase II/III studies have shown encouraging outcomes following ICT in patients with LA-NPC [5, 8, 14, 15, 26, 27]. Platinum drugs are the most widely used agent for both ICT and CRT in patients with NPC. Lobaplatin is a third-generation platinum agent that shows less gastrointestinal, auricular, liver and renal toxicity than cisplatin or carboplatin [18]. Lobaplatin combined with or without other reagents has shown encouraging or equivalent anti-tumor efficacy but with less toxicity in squamous cell carcinoma than cisplatin [23, 28, 29]. Notably, lobaplatin combined with docetaxel also showed anti-tumor effects in recurrent or metastatic NPC [24, 30]. However, as the anti-tumor efficacy of lobaplatin as a first-line strategy in LA-NPC was not determined, we conducted the current prospective phase II clinical trial.

In previous studies, ORRs following the addition of different ICT regimens with and without CRT in LA-NPC were 79.4–90.0% and 85.3–100%, respectively, after ICT and a full course of treatment [5, 8, 17, 26]. In the present study, the ORR was encouraging after two cycles of ICT and CRT compared with a previous study in which patients with LA-NPC received cisplatin-5-fluorouracil ICT followed by cisplatin-radiotherapy (88.1% vs. 79.4%, 100% vs. 85.3%, respectively) [31]. Moreover, the 3-year OS and PFS rates of our study are also comparable with previous studies (94.9% vs. 80.0–95.0% and 86.4% vs. 54.0–89.9%) [5, 8, 17, 26, 31]. Although there is a bias in comparing our study with previous reports, we were able to estimate lobaplatin’s activity as a first-line reagent in LA-NPC. However, randomized controlled studies are needed to compare the efficacy of lobaplatin and other reagents as a first-line systemic treatment for LA-NPC.

In this study, 74.6% of the patients received allocated cycles of chemotherapy, and the remaining participants received two or three cycles of chemotherapy mainly as a result of grade 3–4 thrombocytopenia, which has been reported as the major dose-limiting toxicity of lobaplatin [20]. Similarly, thrombocytopenia was also the most frequent grade 3–4 toxicity in our study. However, both thrombocytopenia and other hematological toxicities could be cured using hematopoietic colony-stimulating factor, which was acceptable. No grade 3 or 4 nausea or vomiting was observed in our study, and the rate of this complication appeared to be much lower than that following cisplatin-5-fluorouracil ICT treatment (0% vs. 23.5%) [31]. As lobaplatin has no or limited renal toxicity, a high-volume fluid infusion is not required during chemotherapy, which could shorten the inpatient period and improve patient compliance. Moreover, the lack of requirement for fluid infusion would allow patients with renal or cardiac dysfunction to receive this treatment, thus expanding the potential population with LA-NPC who could benefit from systemic chemotherapy.

A main limitation of this study is that it was not blinded. Neither the investigators nor the patients were blinded, which might have introduced observation bias from the investigators and psychological bias from the patients. Moreover, it was a single-arm trial from a single institution; therefore, the superiority of lobaplatin over other platinum reagents for treatment of LA-NPC could not be defined, and the efficacy of lobaplatin in other non-endemic areas is also needed. Additionally, six patients were lost to follow-up by 3 months after treatment, leaving the lost-to-follow-up rate (about 10%) higher than in similar studies [7, 32], which might have resulted in bias when forming conclusions. Additionally, long-term follow-up is required to document the long-term efficacy and late toxicities of lobaplatin in patients with LA-NPC.

Altogether, lobaplatin showed promising anti-tumor activity with tolerable toxicity when used as a first-line treatment strategy in patients with LA-NPC; however, it might not be the best choice for patients with a low reserve of bone marrow, such as patients over 60 years old. Moreover, lobaplatin has no crossover drug resistance with other platinum reagents [18] and therefore can be used as a substitute in patients resistant to other platinum-based agents. However, further randomized controlled trials are needed to determine the anti-tumor efficacy of lobaplatin compared with other chemotherapeutic reagents in patients with LA-NPC and to help define the best subpopulation that will achieve maximum survival benefits with this drug.

## Conclusions

We reported the short-term results produced by administering lobaplatin-5-fluorouracil ICT followed by lobaplatin-RT in patients with LA-NPC. A multi-center phase III randomized controlled trial is currently being undertaken by our group and collaborators to determine the efficacy of lobaplatin-5-fluorouracil followed by lobaplatin-RT vs. cisplatin-5-fluorouracil followed by cisplatin-RT in patients with LA-NPC. At the time of this writing, the patients in this trial were still undergoing follow-up.

## Abbreviations

CI: Confidence interval
CRT: Concurrent chemoradiotherapy
DM: Distant metastasis
DMFS: Distant metastasis-free survival
ICT: Induction chemotherapy
LA-NPC: Locally advanced NPC
LR: Local recurrence
LRFS: Local recurrence-free survival
NPC: Nasopharyngeal carcinoma
ORR: Objective response rate
OS: Overall survival
PFS: Progression-free survival
RT: Radiotherapy
